# Jasmonic Acid Enhances Pomegranate Resilience to Drought: Insights Into Leaf and Fruit Traits

**DOI:** 10.1002/fsn3.70695

**Published:** 2025-08-04

**Authors:** Waad S. Faizy, Ahmed Alsawaf, Faris Al‐Zuhairi, Zeyad Amer Mustafa, Marwan Abdullah Sanam, Heidar Meftahizade

**Affiliations:** ^1^ Department of Plant Production Techniques, Agricultural Technical College Northern Technical University Mosul Iraq; ^2^ Department of Desertification Control Technologies College of Agricultural Technology Northern Technical University Mosul Iraq; ^3^ Ministry of Education, Salah El‐Dein Education Directorate Tikrit Iraq; ^4^ Department of Horticultural Sciences, Faculty of Agriculture & Natural Resources Ardakan University Ardakan Iran

**Keywords:** anthocyanins, *F*_V_/*F*_M_, growth regulators, methyl jasmonate, stress adaptation, total soluble solids, water stress

## Abstract

Drought stress (DS) in arid and semi‐arid ecosystems poses a significant barrier to pomegranate cultivation, profoundly affecting fruit quality and agricultural productivity. The primary objective of this study was to investigate the effects of DS at levels of 100%, 70%, 40%, and 20% field capacity (FC), and the foliar application of Jasmonic acid (JA) at concentrations of 0, 5, 25, and 50 μM, on the physiological and biochemical traits of pomegranate leaves and fruits over two consecutive years. The evaluated traits included relative water content (RWC), membrane stability index (MSI), chlorophyll index (SPAD), photochemical efficiency of photosystem II (*F*_V_/*F*_M_), hydrogen peroxide (H_2_O_2_), and superoxide dismutase activity (SOD) in leaves, as well as total phenolic compounds (TPC), total antioxidant capacity (TAC), anthocyanins, total soluble solids (TSS), and weight loss in pomegranate fruits. The results demonstrated that DS, particularly at 20% field capacity, significantly reduced RWC, MSI, SPAD, and *F*_V_/*F*_M_, whereas increasing H_2_O_2_ and SOD levels. Jasmonic acid application effectively mitigated these adverse effects, with higher concentrations (25 and 50 μM) markedly improving the plant's physiological stability. In fruits, both DS and JA application enhanced TPC, TAC, anthocyanins, and TSS. Conversely, in fruit, DS increased weight loss, which was less pronounced during fruit sprayed with JA. The beneficial effects of JA, especially in higher JA concentrations, were also observed in both years, mainly under severe DS (FC40 and FC20), highlighting its crucial role in the improvement of mechanisms related to plant tolerance under water deficit. These results accentuate the effectiveness of JA in improving the physiological and biochemical responses of pomegranate under DS and provide an affordable alternative for sustainable management of crops in arid and semi‐arid areas.

## Graphical abstract

The application of JA, particularly at concentrations of 25 and 50 μM, effectively counteracted these adverse impacts by bolstering antioxidant defenses, maintaining physiological stability, and enhancing fruit quality. JA notably alleviated oxidative stress and improved fruit qualitative characteristics, thereby fortifying plant resilience under water‐limited conditions.

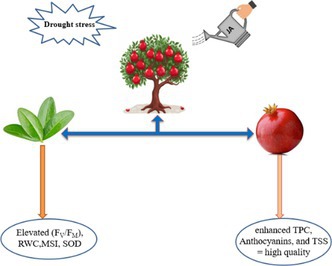

## 1 Introduction

The pomegranate tree ( *Punica granatum* L.) is an important horticultural resource in the semi‐arid climate, due to its great economic value and cultural significance (Holland et al. 2009). Known to have high nutritional values, curative properties, and intermediate tolerance to environmental stresses, this species has a high market preference, both nationally and internationally. The fruit rich in bioactive ingredients like anthocyanins, polyphenolics and organic acids is well‐recognized for its role in human wellness and treatment of various chronic diseases (Viuda‐Martos et al. 2010). Pomegranate fruit has a palatable taste and a high antioxidant activity, which makes it a popular ingredient in the food and pharmaceutical industries, albeit its viability as a stable crop depends on the environment (Teixeira da Silva et al. 2013). The pomegranate tree leaves are also involved in metabolic mechanisms and can help in responses to stress: the physiological changes in foliar regimen directly affect the final characteristics of the fruit (Parvizi and Sepaskhah 2015). However, as a fruit‐bearing tree, pomegranate continues to be highly sensitive to environmental constraints, especially cornea drought, causing severe reduction in crop yield in areas afflicted with water shortage (Galindo et al. 2018). Drought stress is a major problem for present‐day agriculture and has a dramatic effect on the development, productivity, and fruit quality of orchard crops, among which pomegranate (Ruiz‐Sánchez et al. 2010). This environmental limitation results in a series of physiological disturbances, such as low relative water content (RWC), stomatal limitation, restricted photochemical efficiency of photosystem II (*F*_V_/*F*_M_), which consequently contribute to decreased chlorophyll levels (SPAD), high production of reactive oxygen species (ROS), proside hydrogen (H_2_O_2_) and impaired of membrane stability index (MSI) (Fathi and Tari 2016; Habibi et al. 2024). In pomegranate, DS restricts vegetative growth, injures fruit quality since decreased total soluble solids (TSS) and stimulates the protective response by enhancing activity of antioxidant enzymes such as superoxide dismutase (SOD) (Parvizi and Sepaskhah 2015). Moreover, drought induces the accumulation of anthocyanins and total phenolic compounds (TPC) in leaves and berries, which are an adaptive response of the plant to oxidative stress (Mellisho et al. 2012). Seriously, damage caused by these stresses can seriously inhibit pomegranate growth, which can then resist these environments for a long period; so, there is a necessity for new ways of rescue from adversities like these. An innovative strategy to manage the destructive influences of DS is the application of plant growth regulators, such as Jasmonic acid (JA), which has been identified as a major player in stress defense (Asghari et al. 2020). As an important plant hormone, JA regulates a sophisticated defense mechanism in plants to adapt to environmental stress (Wasternack and Hause 2013). By stimulating the molecular signaling cascades, JA induces the expression of the genes that are associated with the SOD, strengthening cellular membrane stability, assisting in the osmotic adjustment as well as intensifying the production of secondary metabolites, such as phenolic compounds and anthocyanins, which enables the plants to tolerate in dry scenario (Shan et al. 2015; Asghari et al. 2020). Previous studies in tomato and grape have shown that JA treatment under water‐limiting conditions significantly enhances antioxidant capacity, optimizes photochemical efficiency of photosystem II, and diminishes oxidative stress damage (Bali et al. 2019; Zhang et al. 2020). In addition, JA regulates hormone catabolism that strengthens plant tolerance to adverse conditions and protects important fruit quality parameters including TSS and anthocyanin levels in challenging situations (Wang et al. 2021). However, the detailed effects of JA on pomegranate under DS, especially on the physiological and biochemical characteristics of the leaves and fruits, are still not well understood, which needs to be studied urgently. To meet this goal, this study was conducted to determine the physiological and biochemical responses of pomegranate trees treated with JA at four different concentrations (0, 50, 100, and 150 μM) and exposed to DS under four irrigation levels as follows: full irrigation and water supply at 70%, 40% and 20% FC. This study has been conducted to assess leaf traits (RWC, *F*_V_/*F*_M_, SOD, MSI, and H_2_O_2_ levels) and fruit traits (TPC, TAC, TSS, total anthocyanin content, and weight loss) during 2 years. It is expected that the results reported here will contribute to a greater understanding of the role of JA as a potential exogenous application tool in attenuating the detrimental effects of DS on the pomegranate, which could be useful in improving the management of modern pomegranate orchards in a water‐deficient environment. Moreover, this study will enhance knowledge of pomegranate's defense against DS and the role of JA in the improvement of fruit quality and leaf heath as well.

## 2 Materials and Methods

A 2‐year experiment was conducted on 4‐ and 5‐year‐old pomegranate trees in the experimental orchard of Mosul city, Iraq. Experimental layout. The experiment was established in a factorial design within a randomized complete block design (RCBD) with two factors under study: irrigation levels and JA application. Water stress was applied during 4 months using four gradient levels of irrigation starting from field capacity (FC); full irrigation, 70%, 40%, and 20% FC. After 25 days, JA was foliar‐applied at 0, 5, 25, and 50 μM, three times at 25‐day intervals. Leaf samples were collected at the end of the stress period from fully developed, mature leaves located in the middle part of the shoots. After the completion of the treatments, leaf samples were immediately frozen in liquid nitrogen and stored at −80°C for subsequent laboratory analyses. The soil texture was sandy loam with 1.8 ds m^−1^, pH 7.6, organic matter content 9.0 g kg^−1^ soil, field capacity 25.1%, total nitrogen (N_kjeldhal_) 0.045%, available phosphorus (P) 124 mg^−1^ kg^−1^ soil, and available potassium (K) 238 mg^−1^ kg^−1^ soil. Also, irrigation was done using drip irrigation. The characteristics of irrigation water was pH: 7.5, EC × 10^6^: 720, HCO_3_^−^: 4.3 meq L^−1^, Cl^−^: 2.1 meq L^−1^, Na^+^: 2.1 meq L^−1^, and SO_4_^−2^: 3 meq L^−1^.

### 2.1 Physiological and Biochemical Traits Related to Leaves

#### 2.1.1 Leaf Relative Water

The relative water content of young, fully expanded leaves was determined using the equation: RWC (%) = [(FM − DM)/(TM − DM)] × 100. In this formula, FM denotes the fresh mass of the leaves at the time of collection, TM represents the turgid mass recorded after immersing the leaves in distilled water at 4°C for 24 h in complete darkness, and DM indicates the dry mass, measured after oven‐drying the samples at 75°C for 72 h (Habibi et al. 2024).

#### 2.1.2 SPAD and Photosystem II Maximal Quantum Yield (*F*_V_/*F*_M_)

Chlorophyll concentration index was determined at 10:00 h with the SPAD‐502 on non‐sampled leaves. Measurements were performed on the second fully expanded leaf, randomly selected, with 15 replications per treatment. The SPAD values were measured with a SPAD‐502 chlorophyll meter to take accurate and repeatable measurements.

The maximum quantum yield of photosystem II (*F*_V_/*F*_M_) was estimated using the OJIP protocol as described by Habibi et al. (2024). Young mature leaves of walnut from the middle part of saplings were carefully selected for each experimental treatment. After 20 min in the dark‐adapted condition, chlorophyll fluorescence parameters were recorded using a portable fluorometer (Fluorpen FP 100‐MAX; Photon Systems Instruments, Drasov, Czech Republic).

#### 2.1.3 Membrane Stability Index

Membrane stability index was determined using the measurement of the electrolyte in leaf tissues, and is considered to be a reliable reflection of cellular membrane integrity during stress. Four‐leaf discs were carefully cut from each plant, and they were all submerged in 20 mL of deionized water for this analysis. Both of the samples were then allowed for 24 h to undissolve the major quantity of electrolyte at room temperature. The electric conductance of the solution, expressed in terms of background leak, was determined using a precision conductometer. Afterward, the leaf discs were heat shocked at 100°C for 60 min to measure total electrolyte content, and a final conductivity value was measured. Electrolyte leakage was then calculated using the following formula: MSI = [1 − (Secondary leakage/Initial leakage)] × 100 (Arora et al. 1998).

#### 2.1.4 Hydrogen Peroxide Levels

To determine the concentration of H_2_O_2_ in plant tissues, the method of Alexieva et al. (2001). Extract preparation: A 0.5 g portion of fresh leaf was homogenized in ice‐cold 0.1% TCA to induce cell lysis. This homogenate was then centrifuged at 12,000 rpm for 15 min to collect the supernatant. A clear extract (0.5 mL of the sample) was mixed with an equal volume of 100 μM potassium phosphate buffer (pH 7.0) and 2 mL of 1 M KI solution. Following incubation in the darkness at room temperature for 60 min, the absorbance was read at 390 nm with a spectrophotometer.

#### 2.1.5 Superoxide Dismutase Enzyme Activity

Superoxide dismutase activity was determined according to the method of Dhindsa et al. (1981). Fifty microliters of enzyme extract was mixed with 1 mL of reaction solution containing 50 mM phosphate buffer (pH 7.8), 13 mM methionine, 25 mM NBT, 0.1 mM EDTA, and 50 mM sodium carbonate. To initiate the photochemical reaction, 2 mM riboflavin was added, and the samples were exposed to two 15‐watt fluorescent lamps for 15 min. A reaction without enzyme extract was added as a control reaction in order to calibrate the maximum colorimetric response. The reaction was terminated by switching off the light source and transferring the tubes to a darkness. The absorbance of the samples was recorded at 560 nm using a spectrophotometer. One unit of SOD activity was defined as the quantity of enzyme that inhibits 50% of the NBT reduction relative to the enzyme‐free control. Enzyme activity was expressed as units per milligram of protein, providing a standardized measure of SOD functionality.

### 2.2 Biochemical Traits Related to Fruit

#### 2.2.1 Total Antioxidant Capacity

The total antioxidant capacity of fruit tissues was evaluated using the DPPH assay, as described by Gil et al. (2000). A 1 g sample of fresh fruit material was blended with 10 mL of 80% methanol and homogenized thoroughly. The mixture was incubated at ambient temperature for 30 min, followed by centrifugation at 4°C for 10 min to obtain the supernatant. For the assay, 100 μL of the leaf extract was mixed with 900 μL of a 500 μM DPPH solution prepared in ethanol, vigorously agitated, and incubated in darkness for 30 min. The extract was replaced with methanol as a control. This value was read at 517 nm with the use of a spectrophotometer. The antioxidant activity was calculated in the assay protocol using the following formula: Antioxidant activity (%) = [1 − ((Absorbance of extract‐DPPH mixture − Absorbance of extract‐methanol mixture)/Absorbance of ethanol‐DPPH control)] × 100.

#### 2.2.2 Contents of Total Phenolic Compounds and Anthocyanin

The total phenolic content was calculated in milligrams of gallic acid equivalent per gram of husk tissue (mg g^−1^). Additionally, the average total flavonoid was expressed as mg of rutin equivalents per gram of fruit matter (mg g^−1^) (Jing et al. 2024). The content of anthocyanins was measured according to the method described by Jing et al. (2024). This method allowed the accurate determination of anthocyanin content, which was expressed as milligrams per kilogram of sample (mg kg^−1^).

#### 2.2.3 Total Soluble Solids and Weight Loss

The soluble solids content was measured in this study using digital refractometer. Fresh fruit or plant tissues were homogenized and then centrifuged to remove their juice. With a Pasteur pipette, a few drops of the extracts obtained were also deposited gently on the refractometer's prism and outgoing TSS were respectively read in % of°Brix. Body weight was recorded with a digital balance. The measurements were made on three replicates containing about four similar fruits each. Results are presented as the mean fruit weight in grams per plant.

#### 2.2.4 Statistical Evaluation of Experimental Outcomes

An experiment consisting of four levels of DS and four levels of hormonal concentration was subjected to factorial arrangement within a CRD with three replicates and was conducted in R program. Statistical analysis: Statistically combined data were graphically represented with *p < 0.05 as indicated using the Prism software. To analyze the interrelationship between the traits studied, Pearson correlation coefficients were calculated by the ggplot2 package from R. Associations between physiological variables were assessed using linear regression analyses also provided by the ggplot2 package in R. Phenotypic plasticity (PP) between the stress and control treatments was calculated using the equation: PP (%) = [(Stress value − Control value)/Stress value] × 100. This thorough statistical method guaranteed reliable analysis of the experimental data.

## 3 Results

### 3.1 Relative Water Content

**FIGURE 1:**
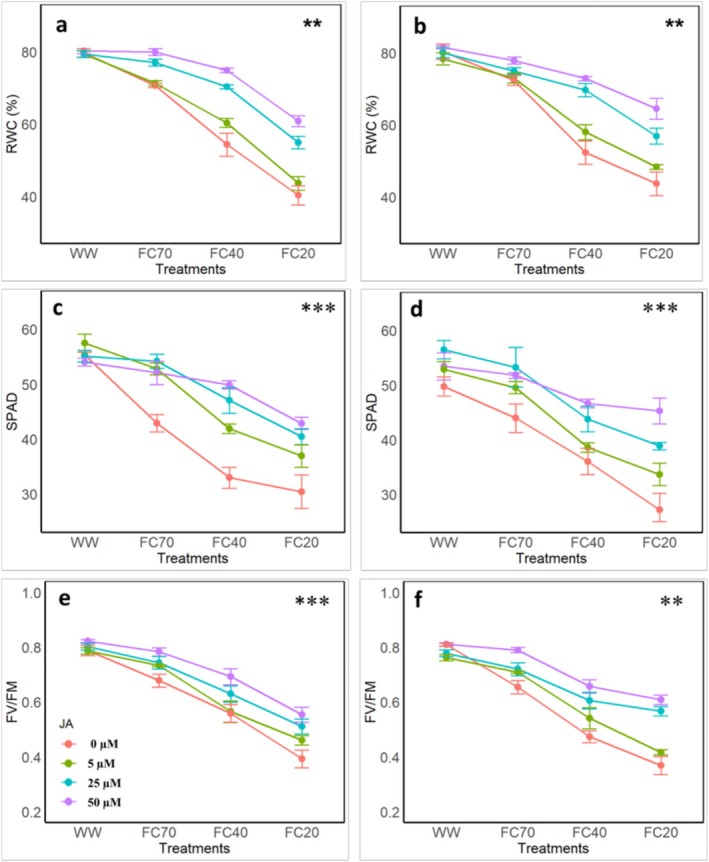
Effects of four irrigation levels (well water [WW], 70% FC, 40% FC, and 20% FC) and four Jasmonic acid (JA) concentrations (0, 5, 25, and 50 μM) on (a, b) the relative water content (RWC), (c, d) SPAD, (e, f) the maximum quantum yield of photosystem II (*F*_V_/*F*_M_) in leaves of pomegranate. The figures a, c, and e show the data after the first experimental year, whereas the figures b, d, and f show the data after the second experimental year. The bars represent the means and standard deviations, whereas different letters represent the significant differences (*p* < 0.01) within the same group.

The results indicated that DS significantly reduced RWC in both years, with the lowest RWC values observed at 20% field capacity (FC20) (Figure 1a,b). In the first year, at the FC40 level, RWC in the control treatment (0 μM) was 31.7%. With the application of JA at concentrations of 5, 25, and 50 μM, RWC decreased to 24.2%, 11.7%, and 6.02%, respectively. At the FC20 level, the highest RWC reduction of 49.3% was recorded in the control treatment (0 μM), whereas with JA application at 5, 25, and 50 μM, the RWC reduction was mitigated to 45.18%, 31.08%, and 23.5%, respectively. Similarly, the effects of the applied treatments on RWC in the second year followed a trend similar to that observed in the first year (Figure 1b).

### 3.2 SPAD Results

The data revealed the significant changes in SPAD values with increasing DS intensity, with JA application modulating these effects, exhibiting distinct patterns in each year (Figure 1c,d). Over the 2 years, DS, particularly at 20% FC, significantly reduced SPAD by 45% and 37.45% in the first and second years, respectively. Conversely, JA application, particularly at higher concentrations (25 and 50 μM), led to a reduction in SPAD across all DS levels. At the FC20 level, the SPAD reduction in treatments with 25 and 50 μM JA was 26.86% and 22.53% in the first year, and 21.85% and 8.98% in the second year, respectively.

### 3.3 Photochemical Efficiency of Photosystem II

The results revealed distinct patterns of photosynthetic responses to DS, modulated by JA, with subtle differences observed between the 2 years. The data indicated that *F*_V_/*F*_M_ exhibited a greater decline with increasing DS intensity in both years (Figure 1e,f).

At the FC40 level in the first year, *F*_V_/*F*_M_ in the control treatment (no JA) decreased by 29.04%. With JA application at concentrations of 5, 25, and 50 μM, the reductions were 28.28%, 20.04%, and 12.01%, respectively (Figure 6a). Under the most severe DS level (20% field capacity, FC20), *F*_V_/*F*_M_ in the control treatment decreased by 50.16%. However, JA application at 5, 25, and 50 μM mitigated the reductions to 41.49%, 35.14%, and 29.62%, respectively. In the second year, the response of relative water content to the applied treatments exhibited a pattern consistent with that observed during the first year (Figure 6c).

### 3.4 Hydrogen Peroxide Content

**FIGURE 2:**
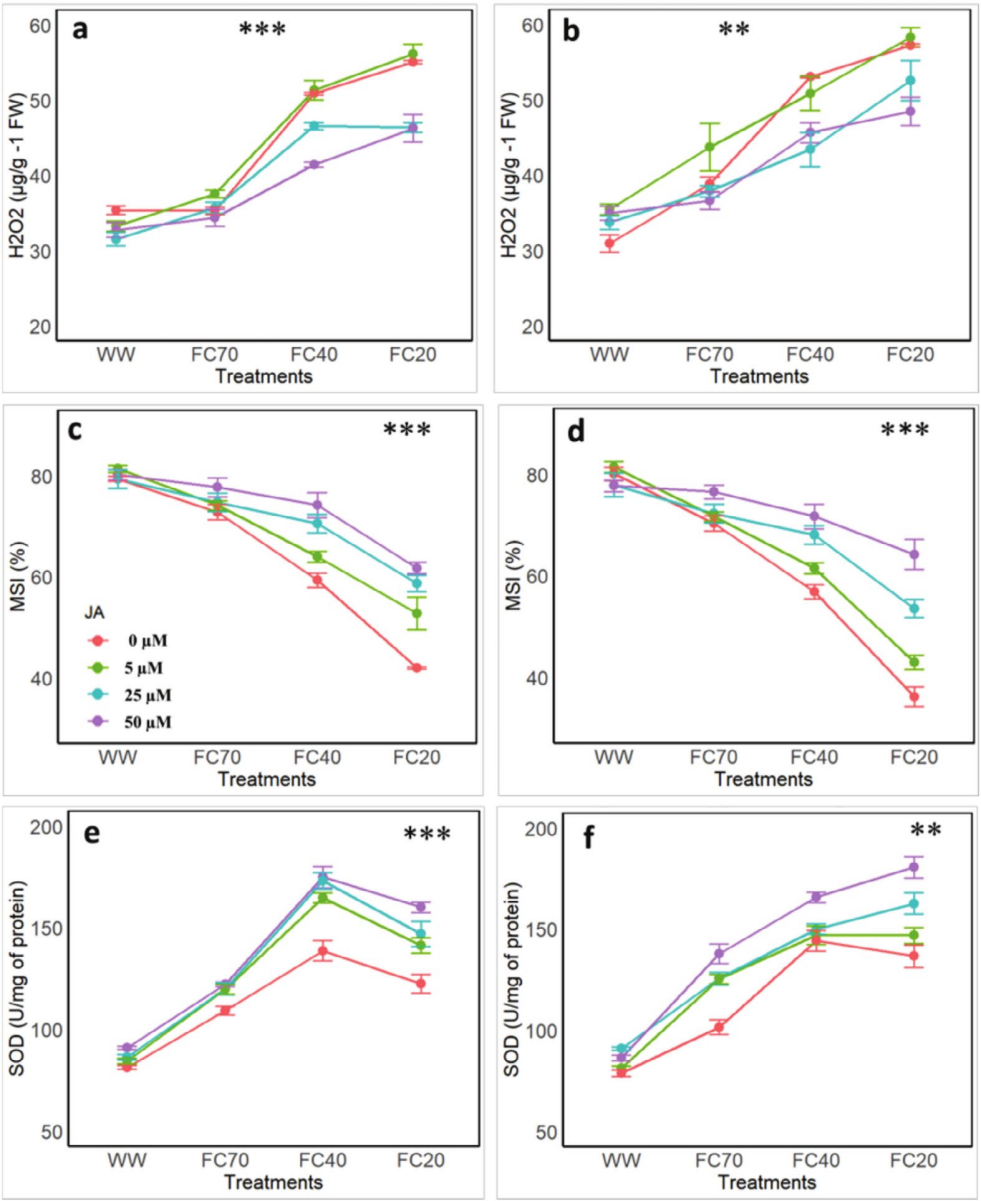
Effects of four irrigation levels (well water [WW], 70% FC, 40% FC, and 20% FC) and four Jasmonic acid (JA) concentrations (0, 5, 25, and 50 μM) on (a, b) Hydrogen Peroxide (H_2_O_2_), (c, d) Membrane Stability Index (MSI), (e, f) superoxide dismutase (SOD) in leaves of pomegranate. The figures a, c, and e show the data after the first experimental year, whereas the figures b, d, and f show the data after the second experimental year. The represent the means and standard deviations, whereas different letters represent the significant differences (*p* < 0.01) within the same group.

The results demonstrated that DS significantly increased H_2_O_2_ levels in pomegranate leaves, with the increase becoming more pronounced as DS intensified. In the first year, the highest H_2_O_2_ increase, amounting to 55.46%, was observed at the FC20 level in the μM treatment (Figure 2a). However, JA application significantly mitigated H_2_O_2_ levels, with reductions to 58.56%, 30.97%, and 30.69% at JA concentrations of 5, 25, and 50 μM, respectively (Figure 6a). In the second year, the trend of H_2_O_2_ changes was more pronounced (Figure 2b). At the FC20 level, the highest H_2_O_2_ increase of 85.57% was recorded in the control treatment (no JA). JA treatments exhibited significant effects in suppressing H_2_O_2_ accumulation, with H_2_O_2_ levels reduced to 69.9% at 25 μM and 56.6% at 50 μM JA concentrations (Figure 6c).

### 3.5 Membrane Stability Index

The membrane stability index (MSI) in pomegranate leaves was evaluated under different levels of DS (100%, 70%, 40% and 20% of FC) and JA applications at concentrations of 0, 5, 25, and 50 μM over two consecutive years (Figure 2c,d). The results demonstrated the significant variations in MSI in response to the intensity of DS and varying JA concentrations. A decline in MSI was observed with increasing DS severity, whereas JA application mitigated this effect. The greatest reduction in MSI was recorded under the WW + FC20 treatment, with reductions of 47.06% and 54.788% in the first and the second years, respectively. However, JA application inhibited further MSI decline. At JA concentrations of 5, 25, and 50 μM, MSI reductions were limited to 33.54%, 26.07%, and 22.2% in the first year, and 46.3%, 33.13%, and 19.7% in the second year, respectively (Figure 6a,c).

### 3.6 Superoxide Dismutase

The experimental results revealed that in the first year, SOD activity levels significantly increased under DS combined with JA application (Figure 2e). Specifically, in the FC40 + 0 μM JA treatment, SOD activity increased by 70.4%. With increasing JA concentrations, further enhancements in SOD levels were observed, with the highest increase recorded in the FC40 + 50 μM JA treatment, reaching 114.94%. However, at the FC20 DS level, SOD levels were lower compared to the FC40 level across all JA concentrations. Significant differences were noted among all applied JA concentrations, with the highest SOD increase attributed to the FC20 + 50 μM JA treatment, amounting to 96% (Figure 6a). In the second year, a similar pattern was observed, albeit with slightly different values (Figure 2f). The results indicated that SOD activity significantly increased under varying DS levels and JA applications. The highest increment of SOD activity was recorded in the FC20 + 50 μM JA treatment, reaching 128.75%, whereas the lowest increment was observed in the FC20 + 0 μM JA treatment, at 73% (Figure 6c).

### 3.7 Total Phenolic Compounds

**FIGURE 3:**
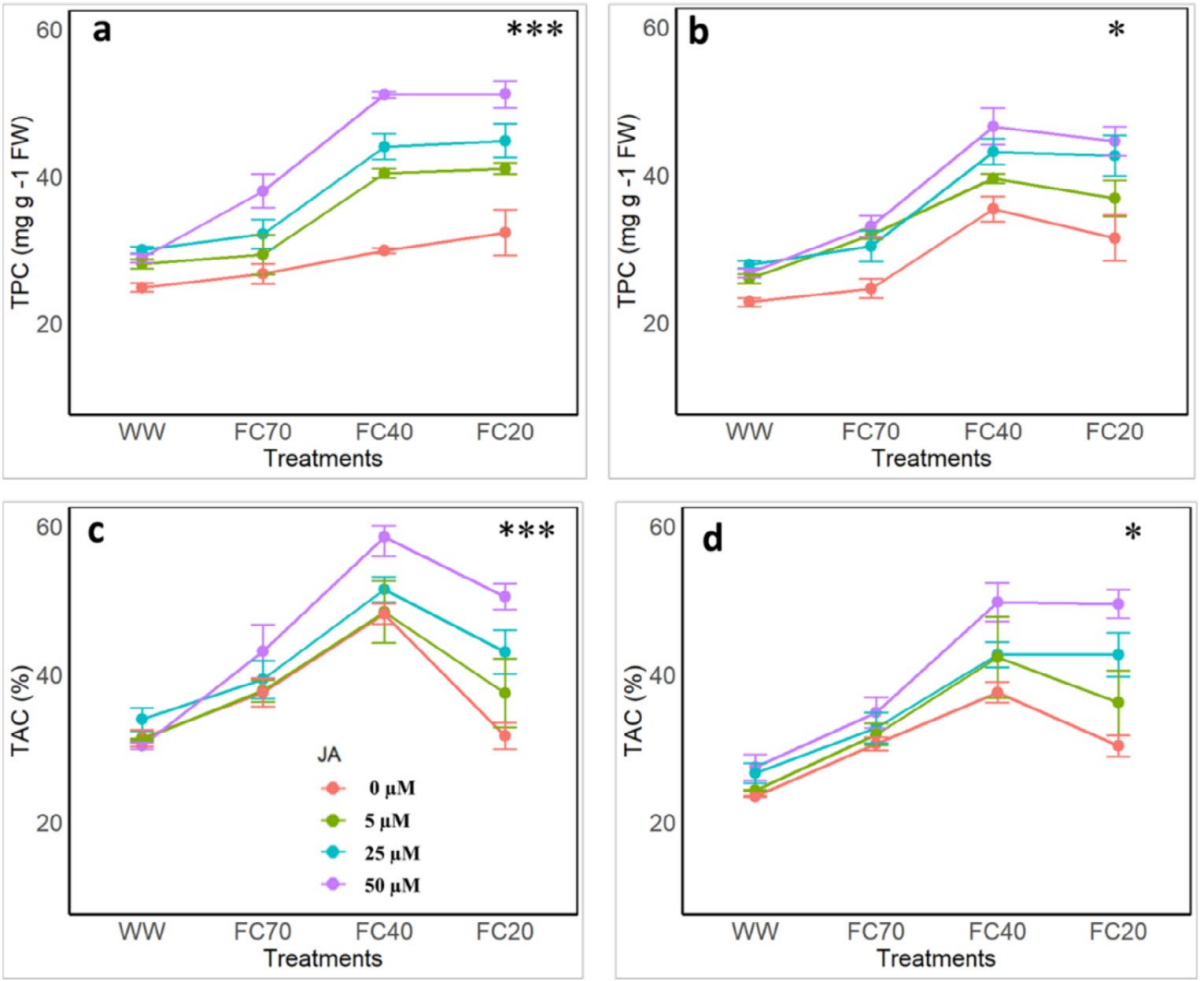
Effects of four irrigation levels (well water [WW], 70% FC, 40% FC, and 20% FC) and four Jasmonic acid (JA) concentrations (0, 5, 25, and 50 μM) on (a, b) total phenolic compounds (TAC), (c, d) total antioxidant capacity (TAC) in pomegranate fruit. The figures a and c show the data after the first experimental year while the figures b and d show the data after the second experimental year. The represent the means and standard deviations while different letters represent the significant differences (*p* < 0.01) within the same group.

The findings of this study revealed that DS and the application of JA had a significant impact on TPC in pomegranate fruits over two consecutive years (Figure 3a,b). In the first year, under full irrigation conditions, the addition of JA at concentrations of 5, 25, and 50 μM increased TPC by 12.7%, 20.5%, and 15.9%, respectively, compared to the control treatment (Figure 6b). At the mild DS level (FC70), TPC in the control treatment increased by 7.5%, whereas with JA application at 5, 25, and 50 μM, TPC values surged to 17.91%, 29.08%, and 52.6%, respectively. Under moderate DS (FC40), TPC in the control treatment increased by 20.2%, but with JA application at 5, 25, and 50 μM, the values elevated to 62.2%, 76.8%, and 105.3%, respectively. At the most severe DS level (FC20), TPC in the control treatment was 29.9%, but with JA application at 5, 25, and 50 μM, the values increased to 64.9%, 80.1%, and 105.4%, respectively (Figure 6b).

In the second year, TPC changed under DS combined with varying JA concentrations and followed a similar trend (Figure 3b).

### 3.8 Total Antioxidant Capacity

The results of this study elucidated the profound impact of DS and JA application on TAC in the pomegranate leaves of the studied plant over two consecutive years (Figure 3c,d). In the first year, the highest TAC values were observed under the FC40 DS level with JA concentrations of 50, 25, 5, and 0 μM, reaching 86.5%, 63.9%, 54.2%, and 53.4%, respectively. However, at the FC20 DS level, TAC values decreased compared to FC40, with the control treatment recording a mere 1%. With JA application at concentrations of 5, 25, and 50 μM, TAC values increased to 19.31%, 36.9%, and 60.9%, respectively (Figure 6d).

In the second year, at the FC40 stress level, TAC in the control treatment was 59.8%, but with JA application at 5, 25, and 50 μM, the values rose to 79.8%, 81.3%, and 111.6%, respectively. At the FC20 stress level, TAC values showed no significant changes across JA concentrations, with values of 53.8%, 81.5%, and 110.7% at 5, 25 and 50 μM JA, respectively. However, in the 0 μM JA treatment, TAC drastically declined to 29.2% (Figure 6d).

### 3.9 Total Soluble Solids

**FIGURE 4:**
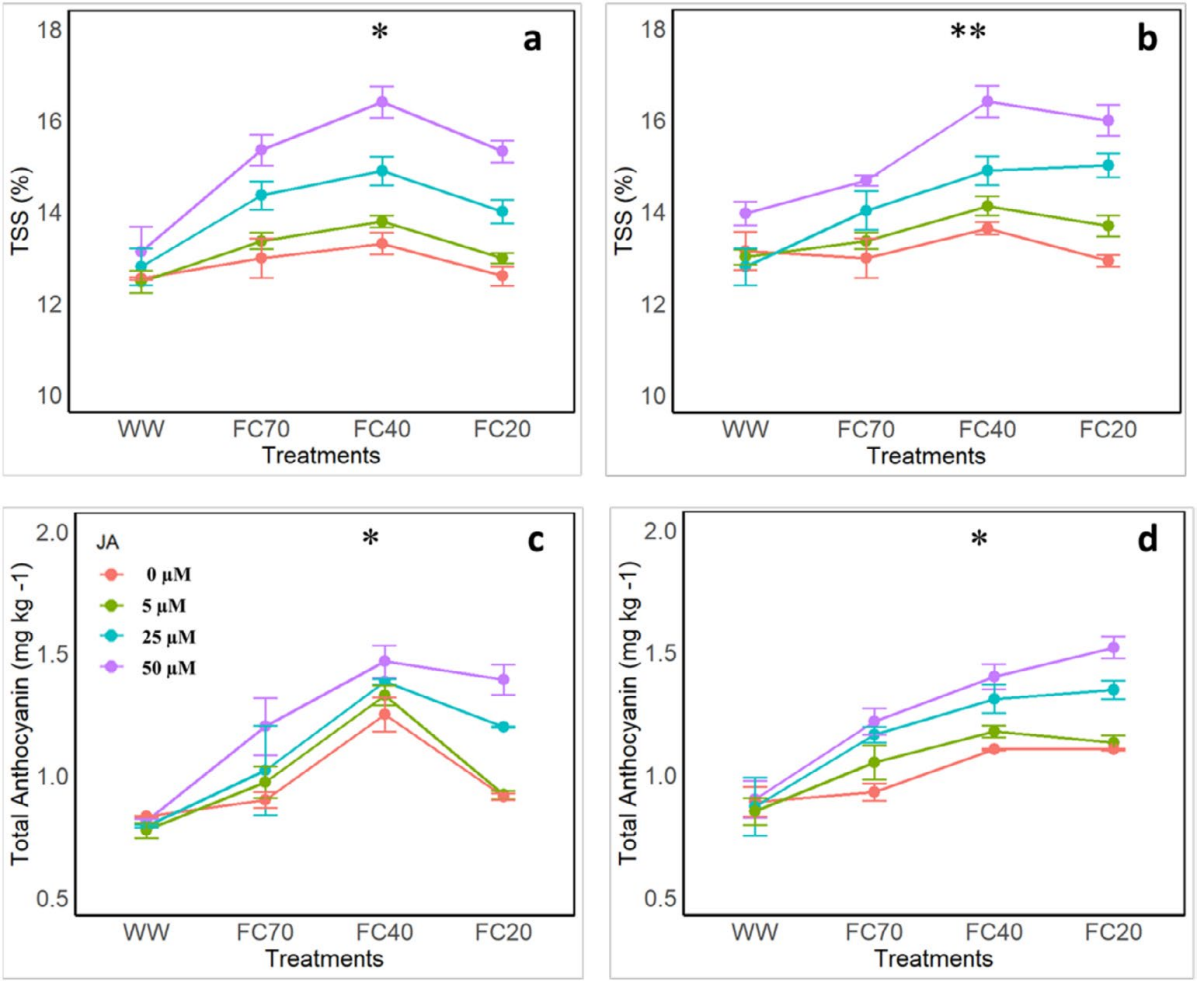
Effects of four irrigation levels (well water [WW], 70% FC, 40% FC, and 20% FC) and four Jasmonic acid (JA) concentrations (0, 5, 25, and 50 μM) on (a, b) total soluble solids (TSS), (c, d) total anthocyanin in pomegranate fruit. The figures a and c show the data after the first experimental year, whereas the figures b and d show the data after the second experimental year. They represent the means and standard deviations, whereas different letters represent the significant differences (*p* < 0.01) within the same group.

Observations from both the first and second years revealed that DS and JA application exhibited similar trends in TSS increment (Figure 4a,b). In the first year, the greatest TSS increase was recorded in the 50 μM JA treatment at the FC40 level, reaching 30.8%. However, at the FC20 level with 50 μM JA, TSS decreased to 22%. In the second year, the highest TSS increases were observed at the 50 μM JA concentration, with values of 24% and 21% at the FC40 and FC20 stress levels, respectively (Figure 6d).

### 3.10 Total Anthocyanin Content

The findings indicated that in the first year, under moderate DS (FC40), the anthocyanin content in the control treatment reached 50.3% (Figure 4c). With JA application at 5, 25, and 50 μM, the values surged to 59.7%, 66.4%, and 76.4%, respectively. At the severe DS level (FC20), the anthocyanin content in the control treatment was notably low at 9.82%, but with JA application at 5, 25, and 50 μM, the values increased to 10.6%, 44.14%, and 67.3%, respectively. In the second year, the anthocyanin content exhibited an increasing trend across all DS levels, with the highest value observed at the FC20 level, reaching 70.5%.

### 3.11 Weight Loss

**FIGURE 5:**
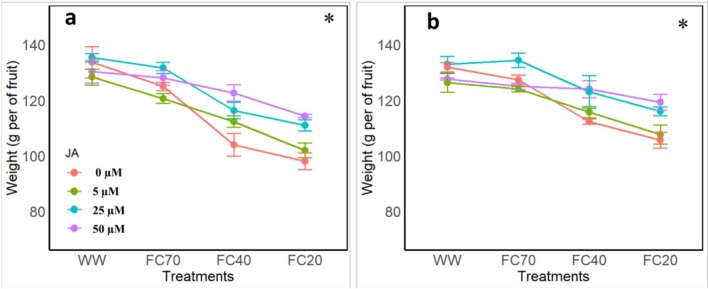
Effects of four irrigation levels (well water [WW], 70% FC, 40% FC, and 20% FC) and four Jasmonic acid (JA) concentrations (0, 5, 25, and 50 μM) on pomegranate fruits weight in the first (a) and the second year (b). The bars the means and standard deviations while different letters represent significant differences (*p* < 0.01) within the same group.

**FIGURE 6:**
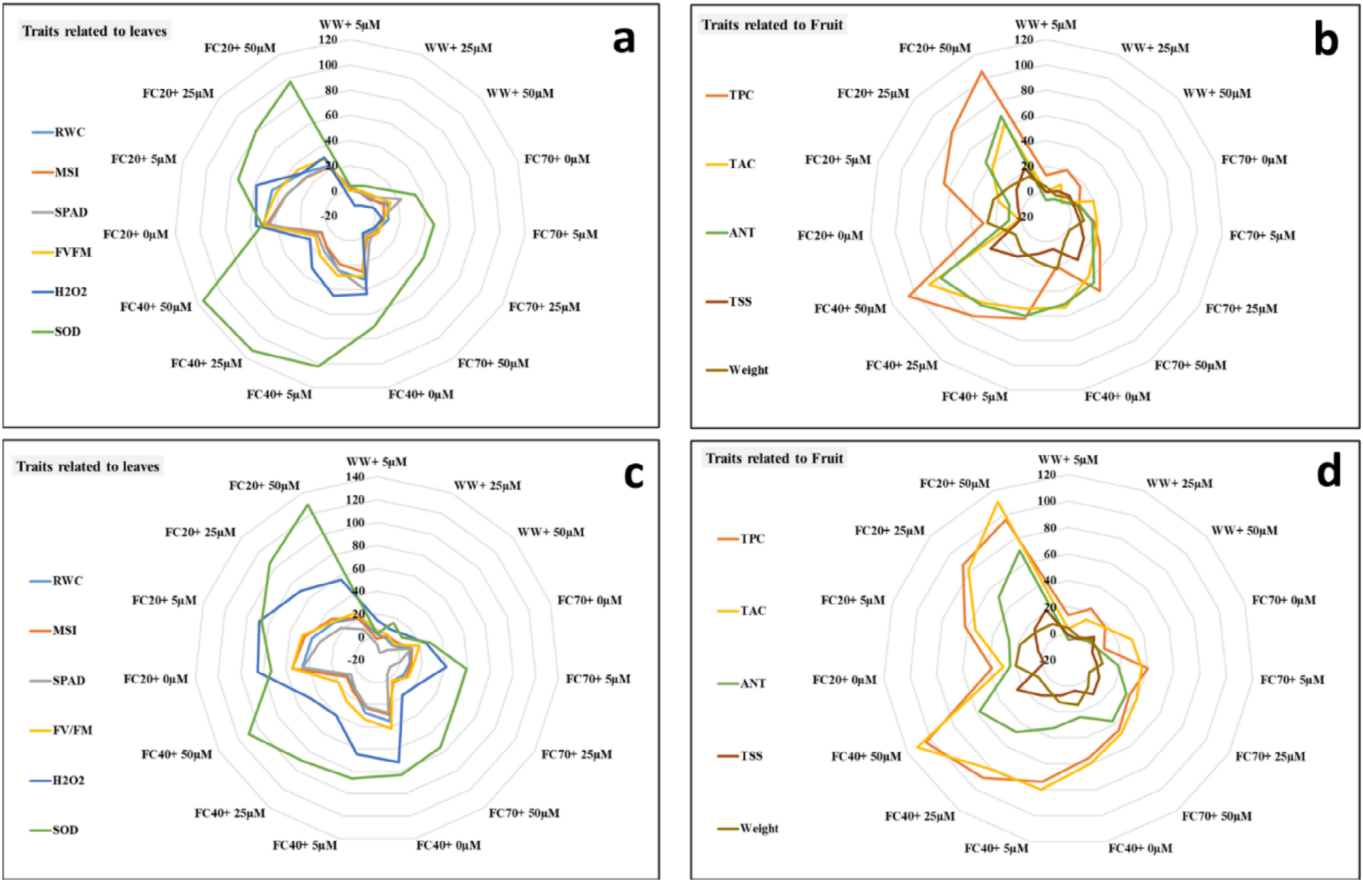
Spider plot showing the percentage change in all phenotypic traits between the control and each treatment. The treatments include four irrigation levels (well water [WW], 70% FC, 40% FC, and 20% FC) and four JA concentrations (0, 5, 25, and 50 μM). Figures a and c present the data related to the pomegranate leaves in the first and the second year, respectively. Figures b and d present the data for the pomegranate fruits for the first and the second year, respectively.

This study investigated the effects of varying DS levels and JA applications on fruit weight in pomegranate over two consecutive years (Figure 5a,b). Overall, fruit weight decreased across all treatments in both years. The greatest reductions were observed under moderate (FC40) and severe (FC20) DS levels in the control treatment, with reductions of 22.1% and 26%, respectively. Conversely, JA application mitigated fruit weight loss under FC40 and FC20 DS levels. At FC40, JA concentrations of 5, 25, and 50 μM reduced weight loss to 15.9%, 12.9%, and 8.2%, respectively. At FC20, JA application at 5, 25, and 50 μM resulted in weight loss reductions of 23.6%, 16.9%, and 14.4%, respectively (Figure 6a).

In the second year, the pattern of fruit weight changes under DS was consistent with the first year (Figure 5b).

### 3.12 Correlation Analysis

**FIGURE 7:**
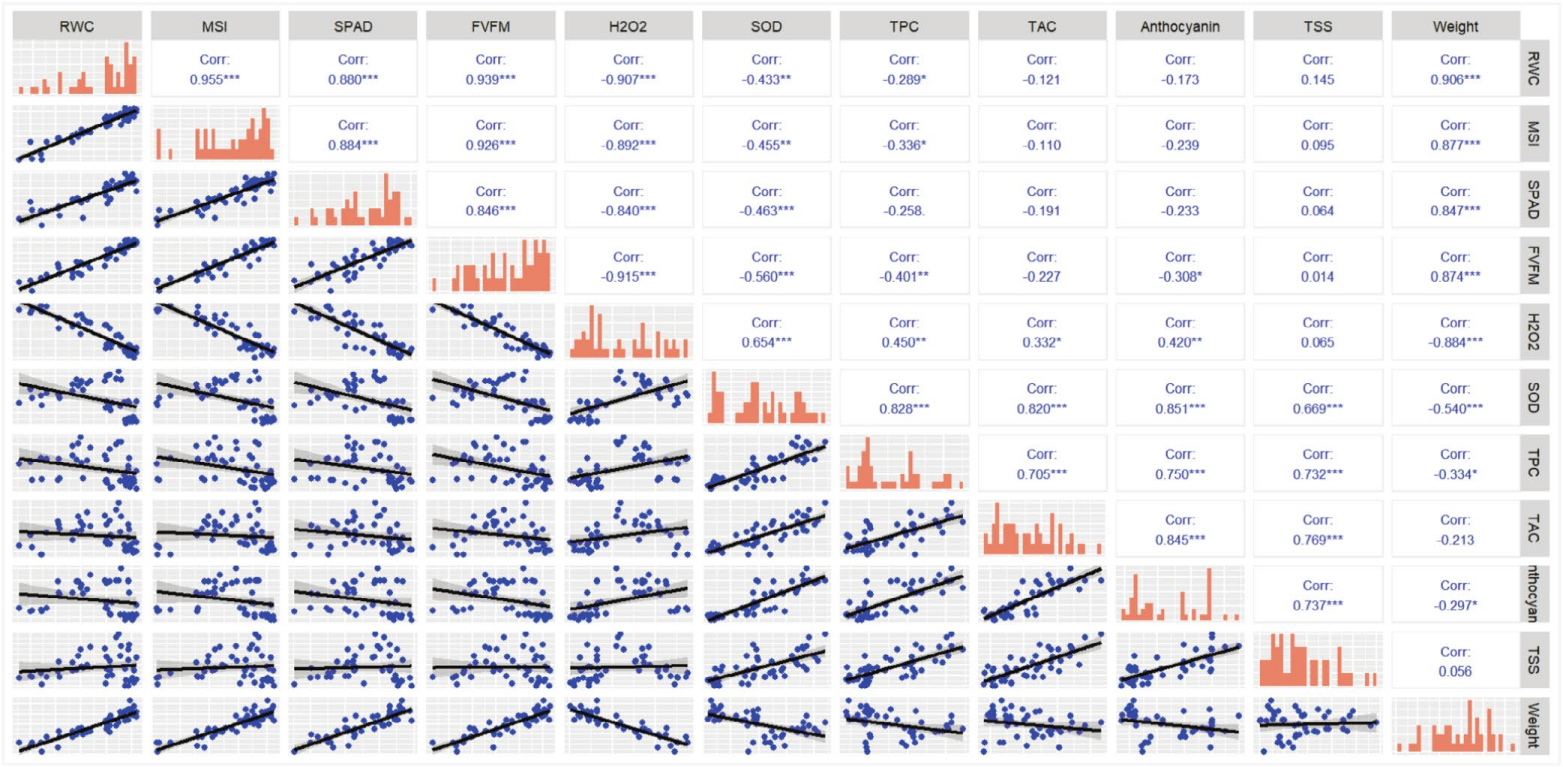
Correlation coefficients of phenotypic traits in pomegranate including leaf traits (RWC, MSI, SPAD, *F*_V_/*F*_M_, H_2_O_2_, and SOD) and fruit traits (TPC, TAC, anthocyanin, TSS, and weight), during stress periods. The numbers in the figure are the Pearson correlation coefficients between the indicators in the first year. Stars in the squares indicate the significance of correlations (*p ≤ 0.05, **p ≤ 0.01, and ***p ≤ 0.001).

**FIGURE 8:**
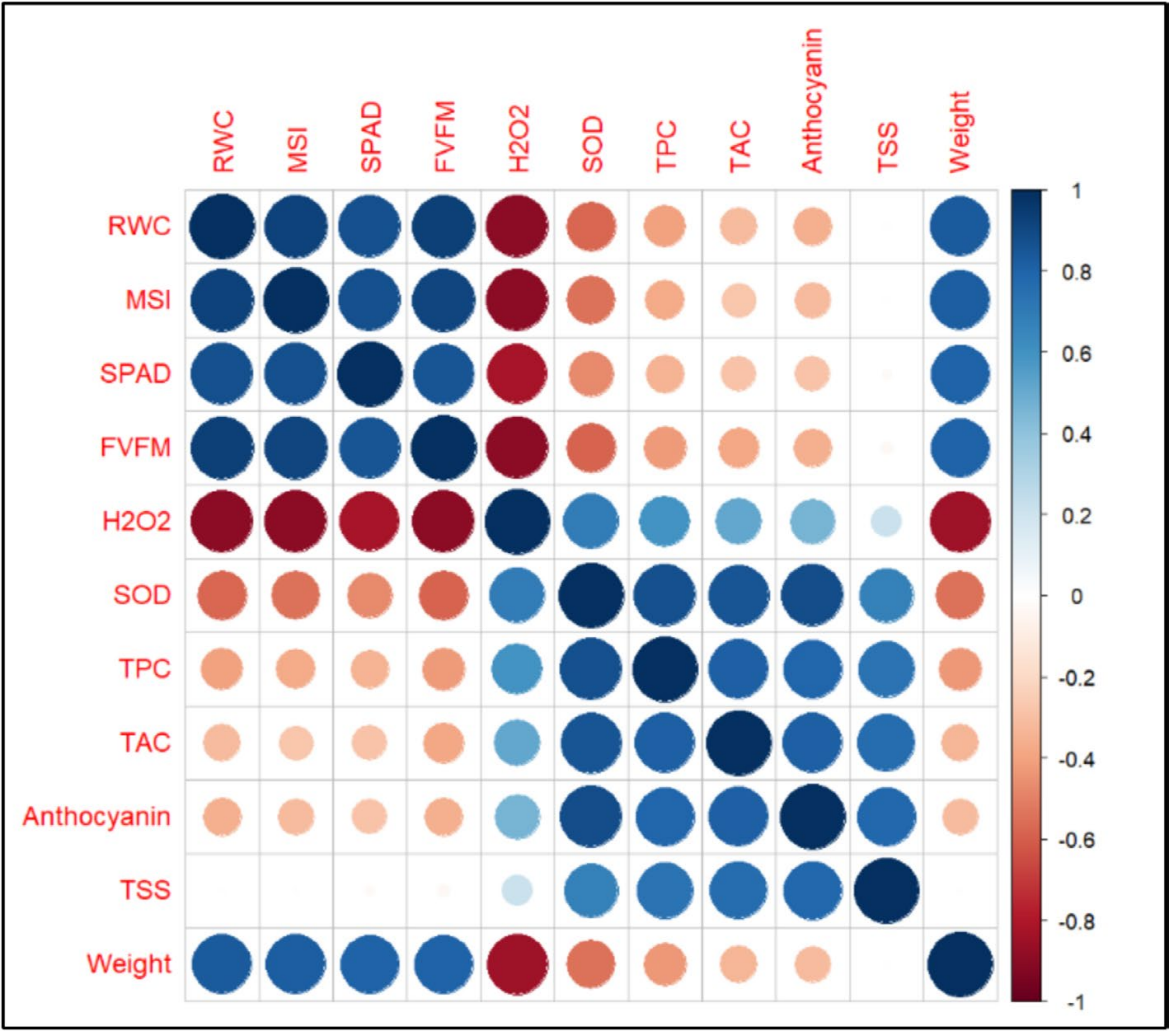
Correlation coefficients of phenotypic traits in pomegranate including leaf traits (RWC, MSI, SPAD, *F*_V_/*F*_M_, H_2_O_2_, and SOD) and fruit traits (TPC, TAC, anthocyanin, TSS, and weight), during stress periods. The color spectrum, from bright blue to bright red, represents highly positive to highly negative correlations. The numbers in the figure are the Pearson correlation coefficients between the indicators in the second year. Stars in the squares indicate the significance of correlations (*p ≤ 0.05, **p ≤ 0.01, and ***p ≤ 0.001).

Correlation analyses were conducted to evaluate the relationships among measured leaf and fruit traits of pomegranate trees across two consecutive years (Figures 7 and 8). The trends in correlation results were generally consistent between the 2 years. The results revealed that the RWC exhibited significant negative correlations with H_2_O_2_ (−0.91 in the first year, −0.89 in the second year) and SOD (−0.43 in the first year, −0.58 in the second year). Conversely, RWC showed significant positive correlations with *F*_V_/*F*_M_ (0.94 in the first year, 0.93 in the second year), SPAD (0.88 in both years), and MSI (0.96 in the first year, 0.93 in the second year). Additionally, RWC in leaves displayed a significant positive correlation with fruit weight, with coefficients of 0.91 in the first year and 0.84 in the second year (Figures 7 and 8).

Furthermore, a strong negative correlation was observed between H_2_O_2_ and other traits (−0.89 in the first year, −0.90 in the second year), whereas a strong positive correlation was found with SOD (0.65 in the first year, 0.70 in the second year). Regarding fruit traits, the results indicated a strong positive correlation between TPC and TAC, with coefficients of 0.71 in the first year and 0.82 in the second year. Similarly, a strong positive correlation was observed between TSS and anthocyanin content, with coefficients of 0.74 in the first year and 0.79 in the second year. Additionally, a significant positive correlation was found between TAC and total antioxidant compounds, with coefficients of 0.85 in the first year and 0.82 in the second year (Figures 7 and 8).

### 3.13 Relationship of Traits

**FIGURE 9:**
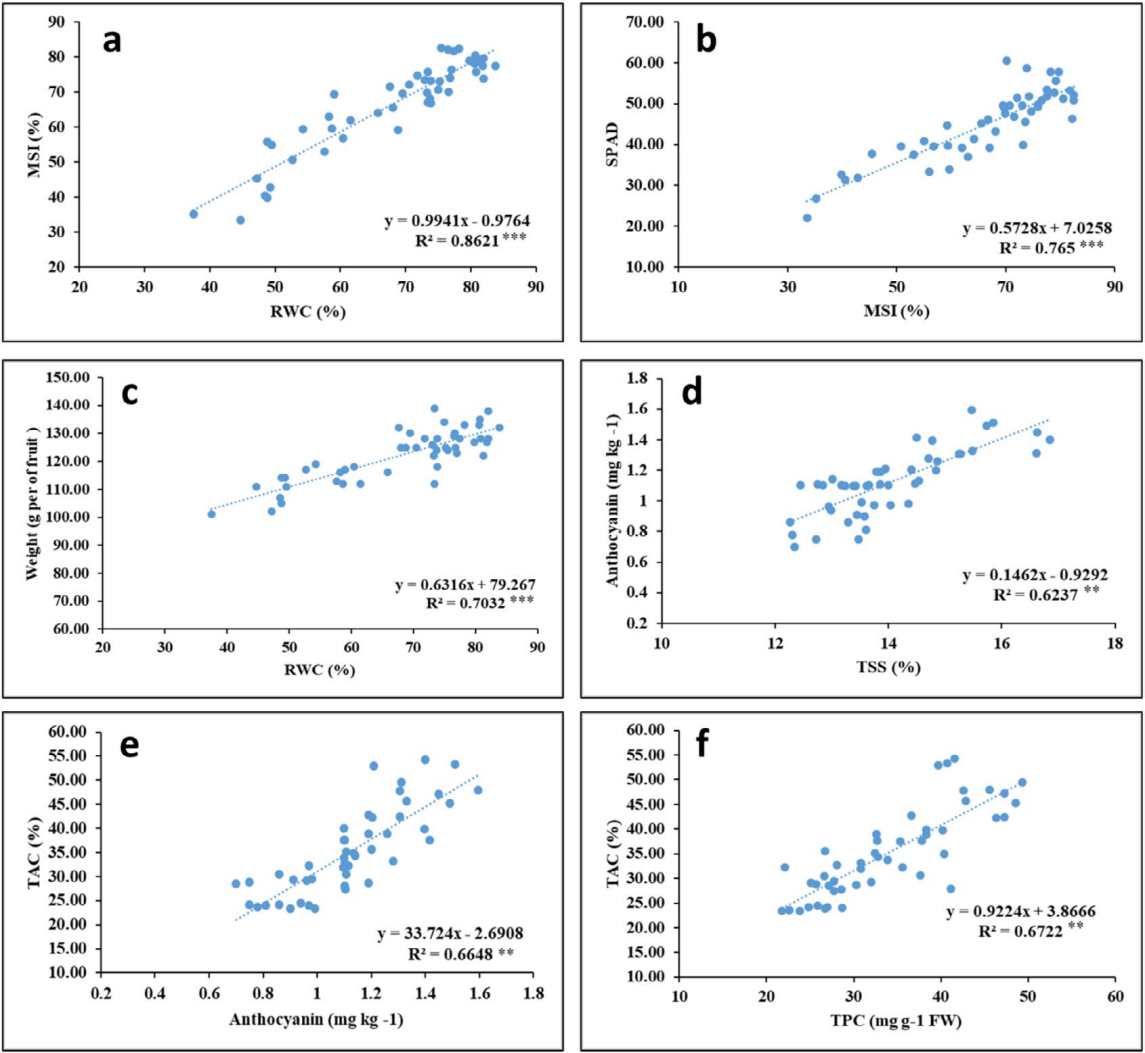
Relationships between (a) relative water contact (RWC) with membrane stability index (MSI), (b) MSI with SPAD, (c) RWC with weight, (d) total soluble solids (TSS) with anthocyanin, (e) anthocyanin with total antioxidant capacity (TAC) and (f) total phenolic compound (TPC) with TAC after interaction irrigation and Jasmonic acid in different levels in the second year. The coefficients of determination (*R* ^2^) of the relationships are given (*p* < 0.01).

Linear regression analyses of the traits measured in this study revealed robust associations among key phenotypic indices. Specifically, the RWC index exhibited a strong relationship with the MSI, yielding a regression coefficient of 0.861 (Figure 9a). Similarly, MSI demonstrated a significant correlation with the SPAD index (0.765) (Figure 9b). Furthermore, a noteworthy relationship was observed between leaf RWC and fruit weight loss, with a coefficient of 0.703 (Figure 9c). In terms of fruit traits, regression analyses underscored a significant association between TSS and anthocyanin content (0.623) (Figure 9d). Additionally, a meaningful relationship was identified between anthocyanin and TAC, with a coefficient of 0.664 (Figure 9e). Moreover, a strong correlation was detected between TPC and TAC, registering a coefficient of 0.672 (Figure 9d).

## 4 Discussion

Drought stress represents a critical constraint on the growth and productivity of fruit trees, particularly in arid and semi‐arid regions. Its detrimental effects are largely exerted through disruptions in key physiological and biochemical processes, significantly impacting both leaf and fruit characteristics.

Our results indicated that DS (most severely at 20% and 40% FC) caused a significant reduction in RWC. This decrease represents an imbalance in plant water status with reduced turgor pressure, indicating the physiological stress induced by water stress. These findings are in agreement with other works already reported in crops, such as olive tree and pomegranate, where RWC has been shown to be a good indicator, reliable and sensitive, of water‐stress response (Bacelar et al. 2007; Rodríguez et al. 2012; Vahdati et al. 2023). In addition, the effect of JA treatment (25 and 50 μM) seemed to mitigate the negative impact of drought by the maintenance of higher levels of RWC. It is possible that these increases could be associated with an increase in the osmotic adjustment and in water uptake through roots, as observed in peach being treated in a similar manner (Sharma et al. 2019). Increasing drought intensity was also linked to successive declines in leaf greenness and *F*_V_/*F*_M_ values, which indicated chlorophyll degradation and photosynthetic efficiency decrease. These results are consistent with previous findings in grapevine (Flexas et al. 2006) and walnut (Habibi et al. 2024), where drought compromised the photosynthetic machinery. Notably, pomegranate JA plants had reduced pomegranate leaf *F*_V_/*F*_M_, and application of foliar JA was able to alleviate the decrease of *F*_V_/*F*_M_ level to a large extent, indicating that JA possibly served a protective role in pomegranate leaves. This might be attributed to the stimulation of genes related to chlorophyll maintenance and the inhibition of oxidative stress, as likewise described for grapevine, citrus (Zandalinas et al. 2017), and pomegranate (Hessini et al. 2019).

In the present study, low irrigation treatments (especially 40% and 20% FC) significantly declined MSI of pomegranate, concomitantly with an increase in H_2_O_2_ content in leaves (Figure 2a–d). These physiological responses are in agreement with studies from other fruit tree species, for example, olive and grapevine, in which restricted water supply resulted in decreases in RWC and SPAD values, with negative effects on turgor maintenance and photosynthetic performance (Flexas et al. 2006; Habibi et al. 2024). Our results further revealed that elevated H_2_O_2_ levels combined with decreased MSI under severe DS reflect the onset of oxidative stress and damage to cellular membranes, which is consistent with previous studies on apple and citrus under similar conditions (Wang et al. 2012; Zandalinas et al. 2017). Interestingly, the foliar application of JA, particularly at higher concentrations, alleviated these negative effects in pomegranate leaves. This mitigation is likely attributed to the activation of SOD and other antioxidant defense mechanisms. JA has been proposed to have a central function in regulating oxidative stress, probably by repressing H_2_O_2_ accumulation and maintaining the stabilized state of photosystem structures under drought‐induced conditions (Asghari et al. 2020). These protective roles are backed up by previous works of Wasternack and Hause (2013) and emphasizing the potential of JA to boost the resiliency of plants in response to environmental stress.

Phenolic compounds may have the key role in determining sensory quality and flavor of these fruits as well as the modulation of plants in response to environmental stress (Habibi et al. 2022). The high antioxidant activities of these compounds are well documented (several studies have found a strong correlation between TPC and TAC) (Derakhshan et al. 2018; Habibi et al. 2023; Shalaby 2024a, 2024b). During DS, the increased production of phenolic compounds is generally an important component of plant protection, together with more efficient TAC. In good agreement with this, we found a high positive association between phenolic content and total antioxidant capacity in the pomegranate fruit (Figure 9f). Additionally, the TPC significantly increased by treating with both DS and JA. Interestingly, studies on other crops, such as tomato (Tao et al. 2022) and blueberry (Wang et al. 2019), have shown that JA can indirectly activate phenolic biosynthetic pathways, thus increasing the antioxidant potential of the fruit. The above observations are consistent with those made in our current study: that is, exogenous JA treatment of pomegranate fruit could also enhance the metabolism of phenolics, presumably helping the fruit to respond to oxidative stress induced by water deficit.

More than colorful anthocyanins, TSS are powerful antioxidant molecules, which certainly contribute to the visual as well as nutritional aspects of the pomegranate fruit (Fan et al. 2016). Treatment with 50 μM JA induced a significant increase in anthocyanins and also maintained TSS both seasons, as shown in the results section. This is in agreement with previous observations in pomegranate. Jasmonic acid is also reported to improve fruit quality by enhancing anthocyanin biosynthesis, which is likely through the up‐regulation of the expression of genes in the secondary metabolite pathways including CHS (chalcone synthase) and UFGT (UDP‐glucose: flavonoid 3‐O‐glucosyltransferase) (Flores et al. 2015; Zhang et al. 2024). Additionally, under DS conditions, plant development can be repressed, which redirects the carbon flux from primary into secondary metabolism. This metabolic change is beneficial for the storage of protective substances that can counteract oxidative damage (Aliabadi Farahani et al. 2009). Such adaptive responses are strengthened by the activation of key biosynthetic enzymes involved in the production of secondary metabolites. Plant growth regulators, including salicylic acid and methyl jasmonate (MeJA), have been shown to enhance the synthesis of antioxidant metabolites, stimulate phenylpropanoid pathways, regulate carbohydrate metabolism, and ultimately improve photosynthetic efficiency. These mechanisms collectively support plant performance under limited water availability (Saharkhiz et al. 2011; Mohammadi et al. 2019; Allagulova et al. 2020).

The current findings clearly indicate that foliar application of JA, particularly at a concentration of 50 μM, plays a pivotal role in enhancing the oxidative stress tolerance in pomegranate. This concentration appears to activate the signaling cascades that stimulate the antioxidant defense system, most notably the SOD enzyme, which in turn leads to a reduction in H_2_O_2_ levels within the leaf tissue, thereby mitigating membrane lipid peroxidation and preserving membrane integrity (Shalaby 2024a, 2024b; Shalaby and Ramadan 2024). Such improvements were also reflected in higher MSI, greater relative RWC, and sustained *F*_V_/*F*_M_, indicating the enhanced physiological performance under drought conditions (Bali et al. 2019). These physiological benefits likely contribute to improved carbon assimilation and nutrient translocation to the developing fruits, culminating in the preservation of TSS and the fruit's overall antioxidant quality (Wang et al. 2021). In addition, JA‐induced antioxidant machinery in the leaves not only strengthens the vegetative tissues against abiotic stress, but also seems to affect the chemical constitution of the fruit (Zhang et al. 2024). The protective metabolites formed in the leaves are probably transferred to the fruit and may contribute to sensory qualities such as flavor, and bio‐functional properties like antioxidant potential (Zhang et al. 2020). Taken together, this research highlights the use of JA as an effective functional biostimulant that can mitigate drought‐induced oxidative damage by strengthening the endogenous defense arena. Its use indirectly benefits the fruit quality through the protection of source tissues, especially under water‐deficit regimes (Zhang et al. 2024). These findings are in keeping with the recent knowledge in other fruits, like grape, citrus, and pomegranate, and point out an encouraging agronomic approach for ameliorating drought stress scenarios in horticultural production systems (Hessini et al. 2019).

Despite the limited number of studies on pomegranate, the available information indicates a beneficial role of certain concentrations of JA and ranges of 50–150 μM in alleviating the reduction in photosynthetic performance under drought (Bali et al. 2019). These results provide a guide for optimal JA dosage in future experimental procedures. However, a better understanding of involved molecular mechanisms needs to be further investigated, particularly at the genomic and metabolomic levels that will contribute to better exploiting the uses of JA in stress management strategies.

## 5 Conclusion

Drought stress had serious adverse effects on the physiological and biochemical characteristics of the pomegranate leaves and fruit. However, the application of JA, particularly at concentrations of 50 μM, effectively counteracted these adverse impacts by bolstering the antioxidant defenses, maintaining the physiological stability, and enhancing the fruit quality. JA notably alleviated oxidative stress and improved fruit qualitative characteristics, thereby fortifying the plant resilience under water‐limited conditions. These results highlight the substantial potential of JA as an innovative and efficient approach for mitigating DS in fruit trees, paving the way for novel strategies to advance sustainable agriculture in arid and semi‐arid environments.

## Author Contributions

**Waad S. Faizy:** conceptualization (equal), funding acquisition (equal), methodology (equal). **Ahmed Alsawaf:** conceptualization (equal), data curation (equal), funding acquisition (equal). **Faris Al‐Zuhairi:** conceptualization (equal), funding acquisition (equal), supervision (equal). **Zeyad Amer Mustafa:** data curation (equal), funding acquisition (equal), investigation (equal), supervision (equal). **Marwan Abdullah Sanam:** conceptualization (equal), formal analysis (equal), investigation (equal), methodology (equal), visualization (equal). **Heidar Meftahizade:** conceptualization (equal).

## Ethics Statement

The authors have nothing to report.

## Consent

The authors have nothing to report.

## Conflicts of Interest

The authors declare no conflicts of interest.

## Data Availability

The authors have nothing to report.

## References

[fsn370695-bib-0001] Alexieva, V. , I. Sergiev , S. Mapelli , and E. Karanov . 2001. “The Effect of Drought and Ultraviolet Radiation on Growth and Stress Markers in Pea and Wheat.” Plant, Cell and Environment 24: 1337–1344.

[fsn370695-bib-0002] Aliabadi Farahani, H. , A. R. Valadabadi , J. Daneshian , and M. A. Khalvati . 2009. “Evaluation Changing of Essential Oil of Balm ( *Melissa officinalis* L.) Under Water Deficit Stress Conditions.” Journal of Medicinal Plant Research: Planta Medica 3: 329–333. 10.5897/JMPR.9000606.

[fsn370695-bib-0003] Allagulova, C. , A. Avalbaev , K. Fedorova , and F. Shakirova . 2020. “Methyl Jasmonate Alleviates Water Stress‐Induced Damages by Promoting Dehydrins Accumulation in Wheat Plants.” Plant Physiology and Biochemistry 155: 676–682. 10.1016/j.plaphy.2020.07.012. 32861034

[fsn370695-bib-0004] Arora, R. , D. S. Pitchay , and B. C. Bearce . 1998. “Water‐Stress‐Induced Heat Tolerance in Geranium Leaf Tissues: A Possible Linkage Through Stress Proteins?” Physiologia Plantarum 103, no. 1: 24–34.

[fsn370695-bib-0005] Asghari, M. M. , M. M. Merrikhi , and B. Kavoosi . 2020. “Methyl Jasmonate Foliar Spray Substantially Enhances the Productivity, Quality and Phytochemical Contents of Pomegranate Fruit.” Journal of Plant Growth Regulation 39: 1153–1161.

[fsn370695-bib-0006] Bacelar, E. A. , J. M. Moutinho‐Pereira , B. C. Gonçalves , H. F. Ferreira , and C. M. Correia . 2007. “Changes in Growth, Gas Exchange, Xylem Hydraulic Properties and Water Use Efficiency of Three Olive Cultivars Under Contrasting Water Availability Regimes.” Environmental and Experimental Botany 60, no. 2: 183–192.

[fsn370695-bib-0007] Bali, S. , P. Kaur , and A. Sharma . 2019. “Jasmonic Acid‐Induced Tolerance to Drought Stress in Tomato Plants Through Enhanced Antioxidant Defense System.” Plant Physiology and Biochemistry 144: 1–10. 10.1016/j.plaphy.2019.09.023.31542655

[fsn370695-bib-0008] Derakhshan, Z. , M. Ferrante , M. Tadi , et al. 2018. “Antioxidant Activity and Total Phenolic Content of Ethanolic Extract of Pomegranate Peels, Juice and Seeds.” Food and Chemical Toxicology 114: 108–111. 10.1016/j.fct.2018.02.023. 29448088

[fsn370695-bib-0009] Dhindsa, R. S. , P. P. Dhindsa , and T. A. Thorpa . 1981. “Leaf Senescence Correlated With Increased Levels of Membrane Permeability and Lipid Peroxidation and Decreased Levels of Superoxide Dismutase and Catalase.” Journal of Experimental Botany 32, no. 1: 93–101. 10.1093/jxb/32.1.93.

[fsn370695-bib-0010] Fan, L. , J. Shi , J. Zuo , L. Gao , J. Lv , and Q. Wang . 2016. “Methyl Jasmonate Delays Postharvest Ripening and Senescence in the Non‐Climacteric Eggplant ( *Solanum melongena* L.) Fruit.” Postharvest Biology and Technology 120: 76–83.

[fsn370695-bib-0012] Fathi, A. , and D. Tari . 2016. “Effect of Drought Stress on Physiological and Biochemical Responses of Fruit Trees: A Review.” Plant Growth Regulation 79, no. 3: 321–335. 10.1007/s10725-015-0139-1.

[fsn370695-bib-0013] Flexas, J. , M. Ribas‐Carbó , J. Bota , et al. 2006. “Decreased Rubisco Activity During Water Stress Is Not Induced by Decreased Relative Water Content but Related to Conditions of Low Stomatal Conductance and Chloroplast CO_2_ Concentration.” New Phytologist 172, no. 1: 73–82. 16945090 10.1111/j.1469-8137.2006.01794.x

[fsn370695-bib-0014] Flores, G. , et al. 2015. “Methyl Jasmonate Treatment Enhances Phenolic Compound Biosynthesis in Grape Berries.” Postharvest Biology and Technology 101: 68–76.

[fsn370695-bib-0048] Galindo, A. , J. Collado‐González , I. Griñán , et al. 2018. “Deficit Irrigation and Emerging Fruit Crops as a Strategy to Save Water in Mediterranean Semiarid Agrosystems.” Agricultural Water Management 202: 311–324. 10.1016/j.agwat.2017.08.015.

[fsn370695-bib-0018] Gil, M. I. , F. A. Tomás‐Barberán , B. Hess‐Pierce , D. M. Holcroft , and A. A. Kader . 2000. “Antioxidant Activity of Pomegranate Juice and Its Relationship With Phenolic Composition and Processing.” Journal of Agricultural and Food Chemistry 48, no. 10: 4581–4589. 11052704 10.1021/jf000404a

[fsn370695-bib-0019] Habibi, A. , S. Sarikhani , M. M. Arab , et al. 2024. “Drought and Heat Stress Interactions: Unveiling the Molecular and Physiological Responses of Persian Walnut.” Plant Physiology and Biochemistry 217: 109237. 39520907 10.1016/j.plaphy.2024.109237

[fsn370695-bib-0020] Habibi, A. , N. Yazdani , N. Chatrabnous , M. Koushesh Saba , and K. Vahdati . 2022. “Inhibition of Browning via Aqueous Gel Solution of *Aloe vera* : A New Method for Preserving Fresh Fruits as a Case Study on Fresh Kernels of Persian Walnut.” Journal of Food Science and Technology 59, no. 7: 2784–2793. 35734107 10.1007/s13197-021-05301-3 PMC9206972

[fsn370695-bib-0021] Habibi, A. , N. Yazdani , M. Koushesh Saba , et al. 2023. “Natural Preservation and Improving Lipid Oxidation Inhibition of Fresh Walnut.” Horticulture, Environment, and Biotechnology 64, no. 1: 133–142.

[fsn370695-bib-0022] Hessini, K. , et al. 2019. “Pomegranate Responses to Water Stress: From Physiology to Molecular Biology.” Plant Physiology and Biochemistry 141: 333–341.

[fsn370695-bib-0023] Holland, D. , K. Hatib , and I. Bar‐Ya'akov . 2009. “Pomegranate: Botany, Horticulture, and Breeding.” Horticultural Reviews 35: 127–191. 10.1002/9780470593776.ch2.

[fsn370695-bib-0024] Jing, S. , C. L. Wang , Y. J. Yang , et al. 2024. “Integrated Transcriptomics and Physiological Parameters Reveal the Husk Browning Difference Between Two Cultivars of Pomegranate Fruit.” Scientia Horticulturae 336: 113413. 10.1016/j.scienta.2024.113413.

[fsn370695-bib-0025] Mellisho, C. D. , I. Egea , and A. Galindo . 2012. “Drought Stress Effects on Antioxidant Enzyme Activity in Pomegranate.” Journal of Plant Physiology 169, no. 12: 1146–1153. 10.1016/j.jplph.2012.03.012.

[fsn370695-bib-0026] Mohammadi, H. , F. Amirikia , M. Ghorbanpour , F. Fatehi , and H. Heshempour . 2019. “Salicylic Acid Induced Changes in Physiological Traits and Essential Oil Constituents in Different Ecotypes of Thymus Kotschyanus and *Thymus vulgaris* Under Well‐Watered and Water Stress Conditions.” Industrial Crops and Products 129: 561–574. 10.1016/j.indcrop.2018.12.046.

[fsn370695-bib-0027] Parvizi, H. , and A. R. Sepaskhah . 2015. “Effect of Drought Stress on Growth and Physiological Characteristics of Pomegranate Trees.” Scientia Horticulturae 197: 519–526. 10.1016/j.scienta.2015.10.014.

[fsn370695-bib-0028] Rodríguez, P. , et al. 2012. “Ecophysiological Responses of Pomegranate ( *Punica granatum* L.) to Water Stress.” Agricultural Water Management 114: 30–38.

[fsn370695-bib-0029] Ruiz‐Sánchez, M. C. , R. Domingo , and J. R. Castel . 2010. “Deficit Irrigation in Fruit Trees: A Review.” Agricultural Water Management 97, no. 10: 1545–1556. 10.1016/j.agwat.2010.05.015.

[fsn370695-bib-0030] Saharkhiz, M. J. , S. Mohammadi , J. Javanmardi , and E. Tafazoli . 2011. “Salicylic Acid Changes Physio‐Morphological Traits and Essential Oil Content of Catnip ( *Nepeta cataria* L.).” Zeitschrift fur Arznei und Gewurzpflanzen 16: 75–77.

[fsn370695-bib-0031] Shalaby, O. A. 2024a. “Moringa Leaf Extract Increases Tolerance to Salt Stress, Promotes Growth, Increases Yield, and Reduces Nitrate Concentration in Lettuce Plants.” Scientia Horticulturae 325: 112654.

[fsn370695-bib-0032] Shalaby, O. A. 2024b. “Using *Bacillus megaterium* as a Bio‐Fertilizer Alleviates Salt Stress, Improves Phosphorus Nutrition, and Increases Cauliflower Yield.” Journal of Plant Nutrition 47, no. 6: 926–939.

[fsn370695-bib-0033] Shalaby, O. A. , and M. E. S. Ramadan . 2024. “Mycorrhizal Colonization and Calcium Spraying Modulate Physiological and Antioxidant Responses to Improve Pepper Growth and Yield Under Salinity Stress.” Rhizosphere 29: 100852.

[fsn370695-bib-0034] Shan, C. , S. Zhang , and D. Li . 2015. “Jasmonic Acid Regulates Antioxidant Defense System and Improves Drought Tolerance in Maize.” Journal of Plant Physiology 184: 1–10. 10.1016/j.jplph.2015.06.009.26151130

[fsn370695-bib-0035] Sharma, M. , et al. 2019. “Jasmonic Acid Modulates Physiological and Biochemical Responses of Peach Trees to Drought Stress.” Scientia Horticulturae 251: 218–228.

[fsn370695-bib-0036] Tao, X. Y. , Q. Wu , J. Y. Li , et al. 2022. “Exogenous Methyl Jasmonate Regulates Phenolic Compounds Biosynthesis During Postharvest Tomato Ripening.” Postharvest Biology and Technology 184: 111760. 10.1016/j.postharvbio.2021.111760.

[fsn370695-bib-0037] Teixeira da Silva, J. A. , T. S. Rana , and D. Narzary . 2013. “Pomegranate Biology and Biotechnology: A Review.” Scientia Horticulturae 160: 85–107. 10.1016/j.scienta.2013.05.017.

[fsn370695-bib-0038] Vahdati, K. , A. Sheikhi , M. M. Arab , S. Sarikhani , A. Habibi , and H. Ataee . 2023. “Cultivars and Genetic Improvement.” In Temperate Nuts, 79–111. Springer Nature Singapore.

[fsn370695-bib-0039] Viuda‐Martos, M. , J. Fernández‐López , and J. A. Pérez‐Álvarez . 2010. “Pomegranate and Its Many Functional Components as Related to Human Health: A Review.” Comprehensive Reviews in Food Science and Food Safety 9, no. 6: 635–654. 10.1111/j.1541-4337.2010.00131.x. 33467822

[fsn370695-bib-0040] Wang, H. B. , Y. Wu , R. P. Yu , C. E. Wu , G. J. Fan , and T. T. Li . 2019. “Effects of Postharvest Application of Methyl Jasmonate on Physicochemical Characteristics and Antioxidant System of the Blueberry Fruit.” Scientia Horticulturae 258: 108785. 10.1016/j.scienta.2019.108785.

[fsn370695-bib-0041] Wang, J. , et al. 2012. “Oxidative Stress and Antioxidant Responses in Apple Under Water Deficit.” Plant Growth Regulation 67, no. 3: 227–235.

[fsn370695-bib-0042] Wang, Y. , X. Zhang , and J. Chen . 2021. “Jasmonic Acid Improves Fruit Quality Under Drought Stress by Enhancing Secondary Metabolite Accumulation.” Postharvest Biology and Technology 171: 111326. 10.1016/j.postharvbio.2020.111326.

[fsn370695-bib-0043] Wasternack, C. , and B. Hause . 2013. “Jasmonates: Biosynthesis, Perception, and Signaling in Plant Stress Response.” Annals of Botany 111, no. 6: 1021–1058. 10.1093/aob/mct067. 23558912 PMC3662512

[fsn370695-bib-0045] Zandalinas, S. I. , et al. 2017. “Antioxidant and Hormonal Responses to Drought Stress in Citrus.” Environmental and Experimental Botany 138: 1–12.

[fsn370695-bib-0046] Zhang, L. , M. Gao , and J. Hu . 2020. “Exogenous Jasmonic Acid Enhances Drought Tolerance in Grapevines by Regulating Antioxidant Enzyme Activity.” Plant Science 291: 110342. 10.1016/j.plantsci.2019.110342.

[fsn370695-bib-0047] Zhang, L. , X. Wu , C. Tian , and R. Schneiter . 2024. “Seasonal Changes in Salicylic and Jasmonic Acid Levels in Poplar With Differing Stress Responses.” Forests 15, no. 11: 1896.

